# New and interesting Orthoptera from the Arabian Peninsula and Socotra

**DOI:** 10.3897/zookeys.679.11967

**Published:** 2017-06-08

**Authors:** Bruno Massa

**Affiliations:** 1 Department of Agriculture, Food and Forest Sciences, University of Palermo, Viale Scienze Bd 4A, 90128 Palermo, Italy

**Keywords:** new records, new species, Oman, *Sphodromerus carapezzanus* sp. n., taxonomy

## Abstract

This paper reports on some interesting taxa recently found in the Arabian Peninsula and the island of Socotra. Among them is a new species of brachypterous grasshopper *Sphodromerus carapezzanus* **sp. n.** (Acrididae: Calliptaminae), described from an isolated area in Dhofar (Oman). A female *Heteracris hemiptera* (Uvarov, 1935) (Acrididae: Eyprepocnemidinae) is reported, with morphological characters which do not fully comply with those of any known subspecies. Two species, hitherto rarely documented, are also reported, *Phaneroptila insularis* Uvarov, 1957 (Tettigoniidae: Phaneropterinae) from Socotra and *Cataloipus thomasi* Uvarov, 1933 (Acrididae: Eyprepocnemidinae) from Oman. *Pycnodictya dentata* Krauss, 1902 (Acrididae: Oedipodinae) is reported from Saudi Arabia, constituting a new record for the country.

## Introduction

The Arabian Peninsula is located between the two wide continents of Africa and Asia, its fauna containing species of Asian and African origin. The Orthoptera fauna of the Arabian Peninsula is quite well known; the list of Ensifera and Caelifera is very long and no less than 190 taxa have been reported (Cigliano et al. 2016). Many contributions have been published in recent years (see Popov 1980, 1981a, 1981b, 1984, 1985, 1997, Ingrisch 1999, Massa et al. 2010, and Buzzetti et al. 2014). In spite of this, there are some isolated regions (like Dhofar in Oman), which are poorly explored. A number of entomological expeditions were carried out by the Museum of Cardiff, and Orthoptera material was also collected by Attilio Carapezza (as part of the Cardiff expeditions), who kindly made it available for study. Among the material identified, a few, but interesting, taxa were found. In this paper these taxa are presented along with some other interesting records from other Arabian localities and the island of Socotra.

## Material and methods

Taxonomical arrangement follows that of Cigliano et al. (2016). Specimens were photographed with a Nikon Coolpix 4500 digital camera, mounted on a Wild M5 Stereomicroscope, and photographs were integrated using the freeware CombineZP (Hadley 2008). Mounted specimens were measured with a digital calliper (precision 0.01 mm). The following measurements were taken (all in mm); body length: dorsal length from the head to the apex of the abdomen (ovipositor excluded in females), pronotum length: length of the pronotum along dorsal median line, pronotum height: maximum height of the pronotum, hind femur: length of hind femur, tegmina: length of tegmina.

### Abbreviations

**BMPC** Collection Bruno Massa, University of Palermo (Italy);

**MSNG** Museo Civico di Storia Naturale ‘G. Doria’, Genoa (Italy).

## Results and discussion

### Tettigoniidae

#### Phaneropterinae

##### Phaneroptila insularis

Taxon classificationAnimaliaOrthopteraTettigoniidae

Uvarov, 1957

Figs 1–3

###### Material examined.

Yemen, Socotra, Wadi Ayhaft 23.I.2014, 12°23'35"N, 53°59'18"E, A. Carapezza (2♂) (BMPC).

###### Remarks.

**Figures 1–3.**
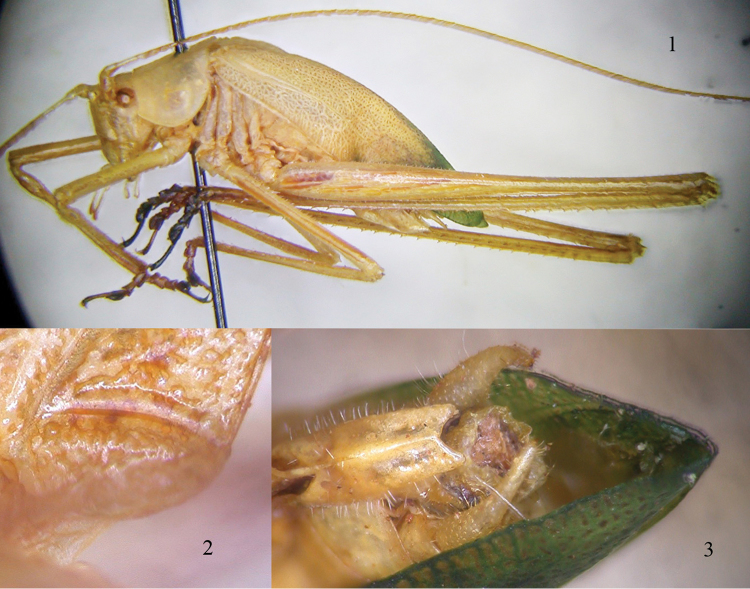
*Phaneroptila insularis* Uvarov, 1957 **1** habitus of male **2** stridulatory file below the left tegmen **3** subgenital plate and cerci of male.

Only one species, endemic to Socotra, is known from this genus. A previous record from this taxon is that of a male holotype, collected on 15 March 1953 along the northern slopes of the Hagghier, at Hijama (Hadiboh Plain) (Uvarov and Popov 1957, Popov 1981a), ca. 15 km away from Wadi Ayhaft. It appears to be a very rare species, where the female still remains unknown. *Phaneroptila insularis* is characterized by the 2^nd^ pair of wings as long as the tegmina (Fig. 1). The stridulatory file, previously undescribed, is 0.6 mm long and consists of approximately 80 evenly spaced teeth (Fig. 2); the subgenital plate of the male is narrow and long, and apically concave. Cerci are robust and incurved (Fig. 3).

### Acrididae

#### Eyprepocnemidinae

##### Cataloipus thomasi

Taxon classificationAnimaliaOrthopteraAcrididae

Uvarov, 1933

Fig. 4

###### Material examined.

Oman, Dhofar, Wadi Ayun (680 m) 18.XI.2016, A. Carapezza (1♂); Oman, Dhofar, Jebel Qamar (650 m) 14.XI.2016, A. Carapezza (1♂) (BMPC).

###### Remarks.

This species is considered to be an endemic taxon to the southern Arabian Peninsula, known only from Dhofar region. According to Popov (1980) it is related to *C. oberthuri* (Bolívar, 1890) from central-east Africa.

**Figures 4–6.**
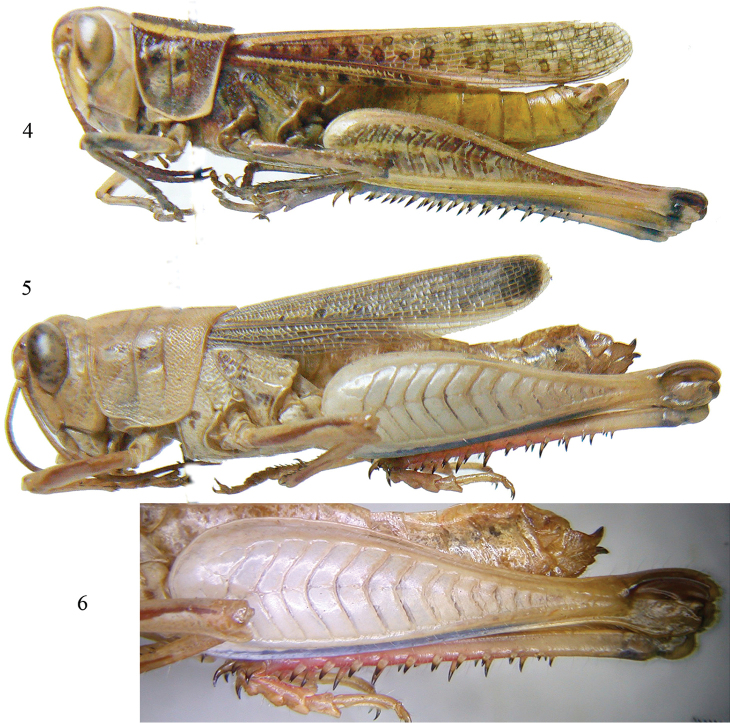
*Cataloipus thomasi* Uvarov, 1933: **4** habitus of male **5** *Heteracris hemiptera* (Uvarov, 1935) ssp.: habitus of female **6** detail of the hind femur of the same.

### Eyprepocnemidinae

#### Heteracris hemiptera

Taxon classificationAnimaliaOrthopteraAcrididae

(Uvarov, 1935)

Figs 5–6

##### Material examined.

Oman, Dhofar, Wadi Mugshail (light trap) 18.XI.2016, A. Carapezza (1♀) (BMPC).

##### Remarks.

Popov (1981b) proposed considering the genus Cyclopternacris Ramme, 1928 as a subgenus of Heteracris Walker, 1870, but Grunshaw (1991), on the basis of male genitalia, decided to synonymize the former. *Heteracris hemiptera aja* was described by Popov (1981b) from a male collected from north-central Saudi Arabia (Shammar). It is different from the typical subspecies, described from Yemen, due to its smaller size, shorter antennae (scarcely longer than head and pronotum together), more rounded hind margin of pronotum, more slender hind femur, shorter and broader supra-anal plate and a more attenuate cercus. In addition, the dark transverse fasciae on the hind femora are well expressed on the upper and inner median areas, while the coloration of the lower sulcus is slate-blue instead of red (Figs 5–6). The female of this taxon was previously unknown. Measurements of the specimen collected from Dhofar lie within the range of females reported by Popov (1981b) for the subspecies *hemiptera*, the antennae are longer than the head and pronotum combined, the hind femur is not as slender as recorded by Popov (1981b) for the subspecies *aja*, and fasciae on hind femora are inappreciable. However, the typical subspecies has the base of the hind femur red, while in the specimen from Dhofar here discussed the color is blue, tibiae are red and hind tarsus is brown (not purple as in *aja* or red as in *hemiptera*). Taking into consideration the geographical position of Yemen, Shammar region, and Dhofar, it seems possible that the female from Dhofar belongs to an unknown taxon, but due to the lack of a male specimens, it is not being described as yet.

Measurements (in mm). Female. Body length: 30.3; length of pronotum: 7.0; height of pronotum: 6.0; length of tegmina: 16.2; length of hind femur: 18.9; height of hind femur: 4.6.

### Oedipodinae

#### Pycnodictya dentata

Taxon classificationAnimaliaOrthopteraAcrididae

Krauss, 1902

##### Material examined.

Oman, Dhofar, Jebel Qara, Jabal Darabab (1100 m) 16.XI.2016, A. Carapezza (1♀); Oman, Dhofar, Wadi Ayun (680 m) 18.XI.2016, A. Carapezza (1♀); Saudi Arabia, Wadi Jizan 11.VIII.1978, Filipponi (1♂) (BMPC).

##### Remarks.

*Pycnodictya dentata* can be separated from *P. galinieri* (Reiche & Fairmaire, 1849) by its blue hind tibiae (purplish in *P. galinieri*) and sinuated posterior lower angle of the pronotal lobes (not sinuated in *P. galinieri*); the color of the hind wings may vary and is not diagnostic (Popov 1980, Ingrisch 1999, Haggag 2016). Specimens here listed as having reddish hind wings. The presence of this species in central-west Saudi Arabia is being recorded for the first time; it was previously reported from Oman and Yemen.

### Calliptaminae

#### Sphodromerus carapezzanus sp. n.

Taxon classificationAnimaliaOrthopteraAcrididae

http://zoobank.org/05EA1E6D-7E89-475A-BEFF-B8FA0AD9DF28

Figs 7–12 , 13–17

##### Material examined.

Oman, Dhofar, Wadi Ayun (680 m) 17°14'53.37"N, 53°53'16.29"E, 18.XI.2016, A. Carapezza (1♂ holotype, 1♀ paratype) (BMPC); (1♀ paratype) (MSNG).

##### Diagnosis.

*Sphodromerus carapezzanus* is very peculiar for its color, brown with tegmina venation being dark and cream spotted. Hind femora with a white base and red outer carinulae, lower genicular lobe white, with the upper part brown, inner face of femora black-reddish, inner tibiae red, outer face of tibiae whitish, spines are black tipped. Carinae of pronotum are distinct in prozona, well visible in metazona.

##### Description.

**Figures 7–12.**
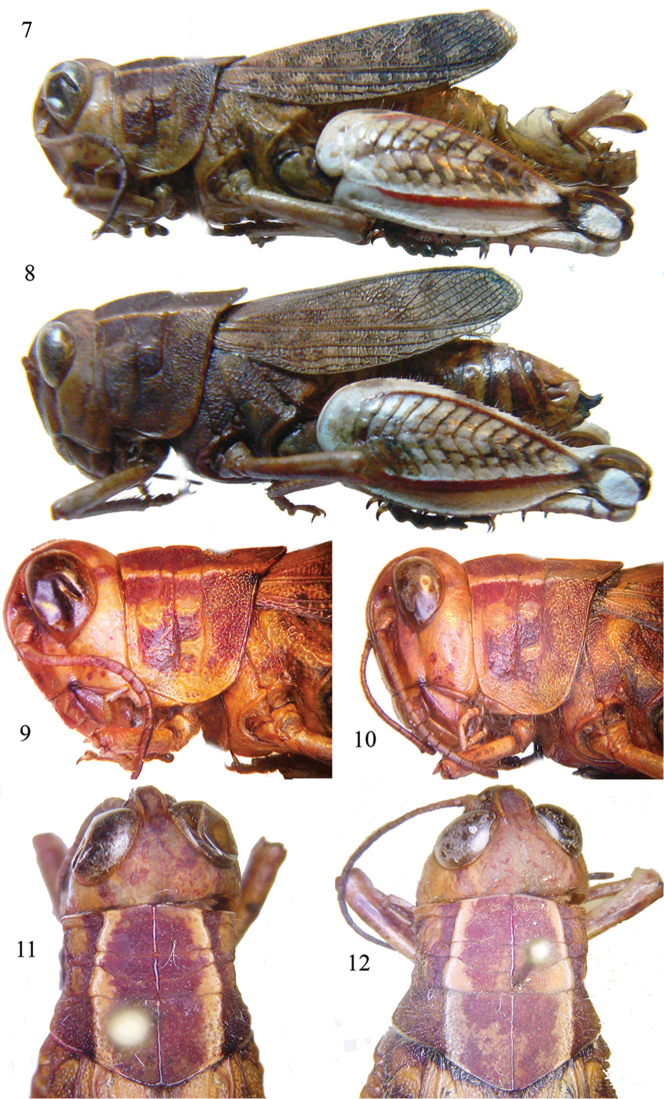
*Sphodromerus carapezzanus* sp. n.: **7** habitus of male **8** habitus of female **9** lateral view of head and pronotum of male **10** lateral view of head and pronotum of female **11** dorsal view of head and pronotum of male **12** dorsal view of head and pronotum of female.

**Figures 13–17.**
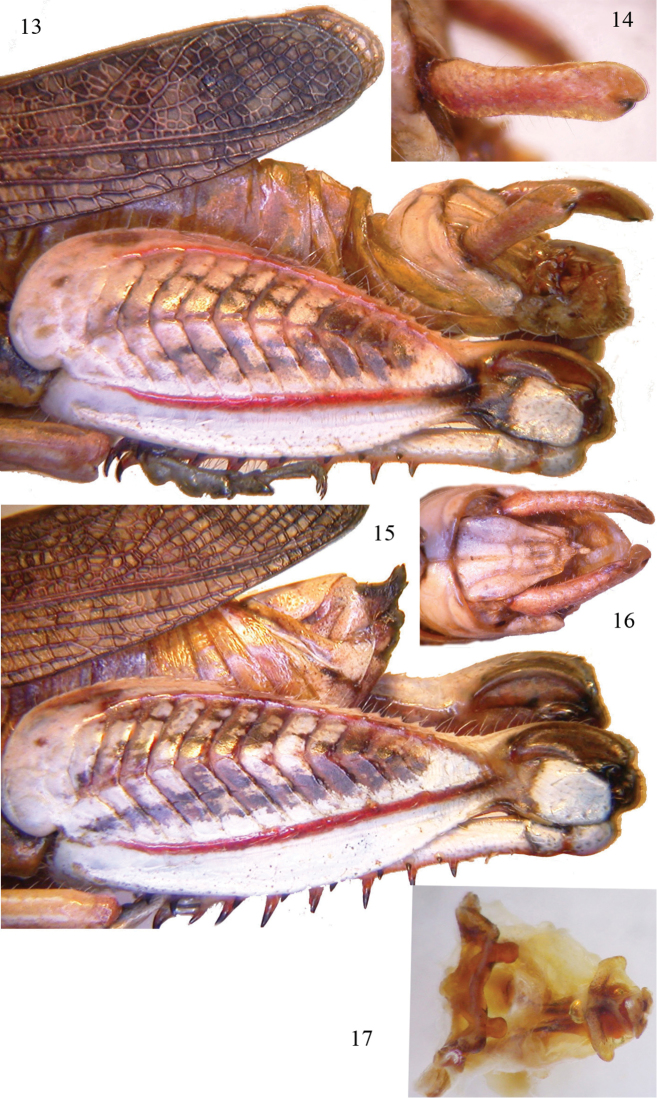
*Sphodromerus carapezzanus* sp. n.: **13** particular of hind femur and last abdominal segments of male **14** left cercus **15** detail of hind femur and last abdominal segments of female **16** dorsal view of last tergites and epiproct of male **17** dorsal view of phallic complex.

Male (Figs 7, 9, 11, 13, 14, 16, 17). Integument finely rugose. Head hypognathous, frontal ridge flat, punctate with margins diverging gradually. Fastigium of vertex depressed longitudinally, narrow, concave, without median carina, margins evident. Frons vertical, slightly convex. Frontal ridge narrow, flat, with a small depression under ocellus. Eyes oval, longer than subocular groove. Antennae filiform, 24 segmented, barely longer than the head and pronotum together (Fig. 7). Pronotum robust, slightly tectiform, lateral carinae distinct, less in metazona, median carina distinct along its entire length, slightly raised, intersected by anterior and posterior sulci (Figs 7, 9, 11). The posterior margin of pronotum obtuse, anterior rounded (Fig. 11), lateral lobes of pronotum with large dots. Prosternal process subconical, with obtuse apex. Hind femora 2.8 times longer than wide, its maximum width behind the middle (Figs 7, 13). Tegmina abbreviated, as long as 2/3 of the abdomen, but shorter than the hind knee (Figs 7, 13). Mesosternal space is 2 times longer than high. Epiproct elongate, with converging lateral margins, margins basally enlarged (Fig. 16), dorsal surface with three longitudinal furrows. Cerci incurved and stout, which is typical of the genus, they are flat with parallel margins, slightly curved and apically divided into two lobes (Figs 13, 14). Subgenital plate conical. Epiphallus broad, ventro-lateral angles projecting, anchorae short, but evident, lophi absent (Fig. 17), ectophallus with a large sclerite, aedeagus with a slender tip (Fig. 17). Hairs sparse in the body, mainly on the legs.

Female (Figs 8, 10, 12, 15). Characters are similar to those of the male, but it is of larger size (see Measurements below), cerci conical, valves of ovipositor short, robust, black-tipped with curved apices (Figs 8, 15).

##### Affinities.

The species is assigned to the Calliptaminae genus *Sphodromerus* Stål, 1873, based on modified male cerci with a single apical tooth and robust femora. The majority of species are described on the basis of their coloration, which seems to be unreliable in similar genera (e.g. *Calliptamus* Serville, 1831) (Uvarov 1922). Three *Sphodromerus* species from the Arabian Peninsula are known to date: (1) *S. pantherinus* Krauss, 1902 from Saudi Arabia, (2) *S. serapis* (Serville, 1838) = *S. scriptipennis* (Walker, 1870) from Arabian Peninsula, and (3) *S. rathjensi* Uvarov, 1936 from Yemen, represented by two subspecies, the nominotypical and *S. r. montanus* Uvarov, 1943. *S. pantherinus* is similar to *S. serapis* from Sinai (Egypt), with tegmina reaching abdominal apex, head and pronotum with blackish spots, inner face of hind femora red, inner hind tibiae red, with inner spines red, outer whitish. *S. scriptipennis* (= *S. serapis*) has tegmina as long as abdomen, hind tibiae yellowish, inner face reddish, with red spines black tipped. *S. r. rathjensi* has hind tibiae and inner face of hind femora blackish-purple, while *S. r. montanus* has these parts pale yellow (see also Ingrisch 1999).

##### Measurements

(in mm). Male. Body length: 23.6; length of pronotum: 4.8; height of pronotum: 5.3; length of tegmina: 11.4; length of hind femur: 12.8; height of hind femur: 4.5. Female. Body length: 28.7–31.9; length of pronotum: 8.3–8.6; height of pronotum: 7.7–8.3; length of tegmina: 16.8–17.9; length of hind femur: 18.6–18.8; height of hind femur: 6.4–6.7.

##### Etymology.

Named for Attilio Carapezza, distinguished Italian heteropterologist, who collected most of the Orthoptera here reported in Oman.

##### Habitat.

**Figure 18.**
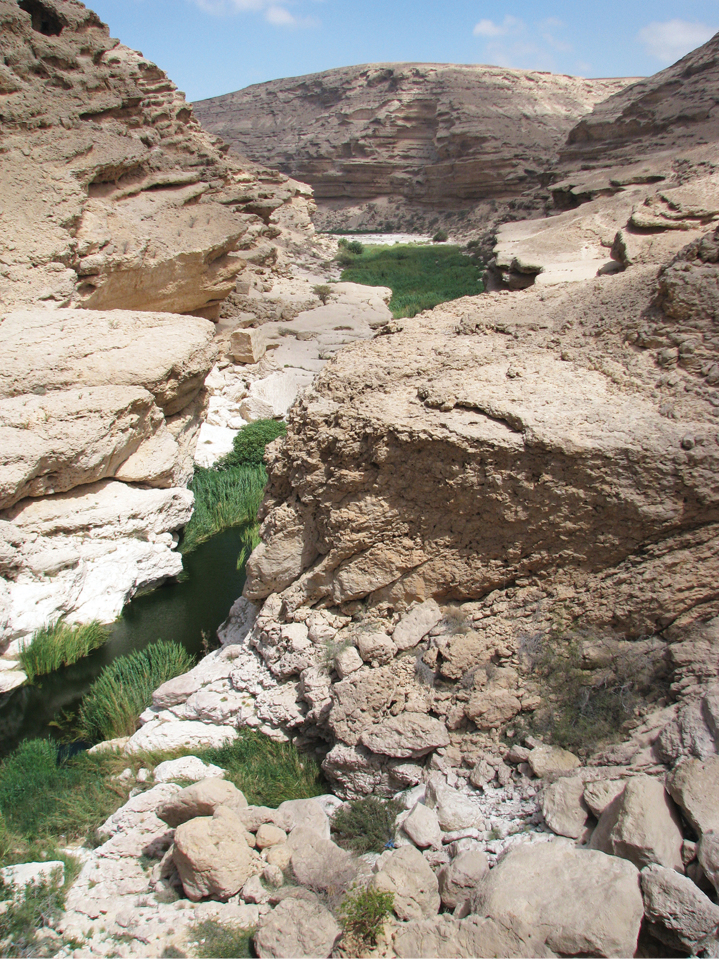
The habitat at Wadi Ayun (Dhofar, Oman) where *Sphodromerus carapezzanus* sp. n. was collected (photo by A. Carapezza).

Wadi Ayun (Arab = Valley of sources) is a very isolated narrow strip of green in an otherwise parched region; at the bottom there are deep pools of flowing blue-green water around sedges and grasses. A rocky desert surrounds it for dozens of kilometers (Fig. 18). Specimens of *Sphodromerus carapezzanus* sp. n. were collected on the ground ca. 200–300 m away from the water.

## Supplementary Material

XML Treatment for Phaneroptila insularis

XML Treatment for Cataloipus thomasi

XML Treatment for Heteracris hemiptera

XML Treatment for Pycnodictya dentata

XML Treatment for Sphodromerus carapezzanus
